# Gender and authorship of publications from Pediatric Acute Lung Injury and Sepsis Investigators (PALISI)

**DOI:** 10.3389/fped.2023.1318690

**Published:** 2023-12-19

**Authors:** Asumthia S. Jeyapalan, Stephanie R. Brown, Mary G. Gaspers, Brittany Haliani, Sapna R. Kudchadkar, Courtney M. Rowan, Shira J. Gertz

**Affiliations:** ^1^ Division of Critical Care Medicine, Department of Pediatrics, University of Miami, Miami, FL, United States; ^2^ Division of Pediatric Critical Care, University of Oklahoma, Oklahoma, OK, United States; ^3^ Department of Pediatrics and Banner Children’s at Diamond Children’s Medical Center, University of Arizona, Tucson, AZ, United States; ^4^ Medical Librarian, Cooperman Barnabas Medical Center, Livingston, NJ, United States; ^5^ Department of Anesthesiology and Critical Care Medicine, Johns Hopkins University School of Medicine, Baltimore, MD, United States; ^6^ Division of Critical Care, Department of Pediatrics, Indiana University School of Medicine and Riley Hospital for Children at IU Health, Indianapolis, IN, United States; ^7^ Division of Pediatric Critical Care, Department of Pediatrics, Cooperman Barnabas Medical Center, Livingston, NJ, United States

**Keywords:** diversity, equity, inclusion, critical care, pediatrics, authorship, publications

## Abstract

**Introduction:**

Pediatric Acute Lung Injury and Sepsis Investigators (PALISI) is a network fostering clinical research to optimize care for critically ill children. We aim to examine the efforts of the PALISI Network to increase gender parity in research, as evidenced by authorship.

**Methods:**

The first and senior authors of all published PALISI articles from 2002 to 2021 were analyzed for gender of presentation. Funding sources, impact factors, professional roles, and location were extracted.

**Results:**

We identified 303 articles, 61 published from 2002 to 2011, and 242 from 2012 to 2021. There were 302 first authors, representing 188 unique individuals, and 283 senior authors, representing 119 unique individuals. Over half (55.6%, *n* = 168) of the first authors were women. More women were first authors from 2012 to 2021 (*n* = 145, 60.2%) as compared to the years 2002–2011 [37.7%, *n* = 23, OR = 2.50 (95% CI: 1.40, 4.45, *p* = 0.002)]. Senior authors were 36.0% (*n* = 102) women, with no change over time. Women senior authors had a higher proportion of women first authors (67.7% vs. 32.4%, *p* = 0.017). No gender differences were noted based on article type or impact factor. The majority of authors came from institutions in the United States. Women had comparatively more NIH and CDC funding but received less funding from foundations and AHRQ.

**Discussion:**

In PALISI publications, first authorship by women has increased over time, such that it now exceeds both the proportion of women pediatric intensivists and women first authors in critical care publications. Senior authorship by women has been stagnant. A multifactorial approach by individuals, institutions, networks, and journals is needed to bring senior women authors to parity.

## Introduction

The proportion of physicians who are women in pediatric critical care medicine, like all specialties, is increasing. According to the AAMC, women accounted for 65% of actively practicing general pediatricians and 49.6% of practicing pediatric intensivists in 2021 (1). In 2022, 57% of those newly board-certified in pediatric critical care medicine were women (2). As the field has grown, so have academic endeavors in research. *Critical Care Medicine (CCM)*, the official journal of the Society of Critical Care Medicine, has noted that women account for 25% of first authors in highly cited articles (3). While pediatrics has had comparatively more women than men complete training since 1989 (4), women remain underrepresented as authors, reviewers, and editors (5). In addition, women have fewer leadership opportunities (6), speaking opportunities (7), and roles on planning committees (8).

The Pediatric Acute Lung Injury and Sepsis Investigators (PALISI) Network was founded in 2002 by Dr. Adrienne Randolph as a collaborative network to promote research in order to optimize the care of critically ill children (9). The volume of rigorous science that has been produced by this group has grown exponentially since its inception, including 12 randomized controlled trials, 10 observational point prevalence and incidence studies, and at least 11 consensus conference topics (9). Thus, we aimed to examine the efforts of the PALISI Network to increase gender equity in research science, as evidenced by authorship.

## Methods

This is a study characterizing the authorship of articles published by the PALISI Network from its inception in 2002 to 31/12/2021. An initial list of eligible articles was requested and received from PALISI leadership. Further searches of PUBMED were conducted using the search terms “pediatric acute lung injury and sepsis investigators” and “PALISI”. The websites of each of the individual PALISI subgroups were examined, and publications, if listed, were extracted. Two authors (ASJ and SRB) voted on articles in the subgroup collections that were not part of the PALISI collections to determine if an article met inclusion criteria. If the vote was split, SJG acted as a tie-breaker. A final extracted article list was compared to a recently generated PALISI list obtained from the current PALISI chair Dr. Neal Thomas with additional verification completed via personal communication with long time PALISI members Drs Neal Thomas Adrinne Randolph and/or Martha A.Q. Curley to determine if an article was a PALISI work product. Notably, no papers from the Pediatric Neurocritical Care Research Group (PNCRG), an affiliated research network, were included.

An analysis of gender of presentation, using gender as a social construct not biological sex, was conducted for both the first and senior authors. Gender of presentation was determined by: (1) personal knowledge of the individual (2) internet searches for academic profiles that used gendered pronouns (3) internet image searches by name and judgment of gender of presentation (4) personal discussion with co-authors of the article. The first author was the first person listed in the author list. Co-first authors were noted, but because they were rare (5%), they were not included in the analysis. The senior author was the last person listed in the author list prior to any listings of research groups or societies. Similarly, senior co-authors were noted but not included in the analysis.

Funding sources and corresponding authors were manually extracted from each article. The professional role was only noted if degrees were listed on the masthead, and only the first degree was noted. Geographical location (state if in the U.S., country if outside of the U.S.) was extracted from the author's information for each article. The impact factor of the journal in the year the article was published was obtained from *Journal Citation Reports* (Clarivate, 2022). For each journal that did not have an impact factor in a given year, the editors were contacted, and verification was obtained that the journal was not indexed.

Study data were collected and managed using REDCap electronic data capture tools hosted at Indiana University (10). Summary descriptive statistics were used and presented as medians with interquartile ranges (IQRs) for continuous variables and counts with proportions (%) for categorical variables. Comparisons were done with chi-squared (or Fisher's exact test, where appropriate) or logistic regression. Trends over time were analyzed with Kendall's Tau. The 20 years included in the study were dichotomized into two 10-year periods: 2002–2011 and 2012–2021. Statistical analysis was completed using STATA for Windows, version 18.0.

As all information used was publicly available, the Cooperman Barnabas Medical Center's Institutional Review Board (22–14, approved 12/05/2022) determined this work to be exempt and not require IRB approval. Procedures were followed in accordance with the ethical standards of the responsible committees on human experimentation and with the Helsinki Declaration of 1975.

## Results

**Figure 1:**
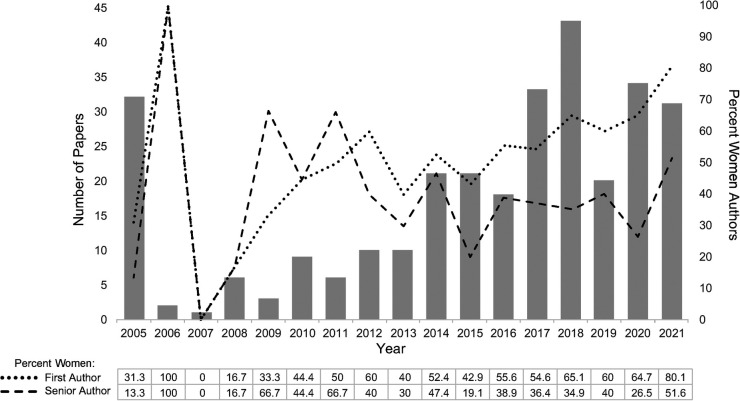
Number of published papers and percentage of women authors per year: the *x*-axis shows the calendar year, with the left *y*-axis having the number of publications and the right *y*-axis the percentage of women authors. First women authors are indicated by the dotted line, and senior women authors by the dashed line. Below each year, the top box shows the percentage of women first authors in that year, and the lower box shows the percentage of senior women authors.

We identified 303 articles that met the inclusion criteria and were work products of PALISI and/or PALISI subgroups published between 2002 and 2021 (Supplementary Material Appendix 1: complete bibliography). One manuscript, the introduction to the Pediatric Acute Lung Injury Consensus Conference, had no authors listed and was therefore excluded, leaving 302 articles for first author analysis and, as there were 19 manuscripts with only one author, 283 for senior author analysis. The number of publications increased steadily over time (T = 0.67, *p* = 0.001) (Figure 1). There were 61 articles published in the first 10 years, between 2002 and 2011, and 241 articles published from 2012 through 2021. The 302 included manuscripts had 318 first authors; 16 articles had two first authors. There were 295 senior authors; eight articles had two senior authors, and one article had three. The majority of articles were clinical research (*n* = 197), with review articles (*n* = 55) and practice guidelines (*n* = 23) constituting most of the remaining article types; there were no differences in author gender noted based on article type.

### First authorship

Of the 302 first authors, 55.6% (*n* = 168) were found to be women and represented 188 unique individuals, of whom 108 (57.5%) were found to be women. The percentage of women first authors increased over time, with 37.7% (*n* = 23) between 2002 and 2011 and 60.2% (*n* = 145) between 2012 and 2021. This difference persisted when looking at unique authors, with 44 unique first authors between 2002 and 2011, where 34.1% (*n* = 15) were women, compared to 144 unique first authors between 2012 and 2021, where 64.6% (*n* = 93) were women. There was an increased odds ratio of a women first author in the second decade, OR = 2.50 (95% CI: 1.40, 4.45, *p* = 0.002). As there were many more manuscripts published in the last decade as compared to the first, we then isolated the manuscripts published from 2012 to 2021. Examining the trends over time between 2012 and 2021, there was a significant increase in the proportion of women first authors (T = 0.58, *p* = 0.025). When further divided into two time periods, from 2012 to 2016, 50.0% (*n* = 40/80) of the manuscripts had women first authors, and from 2017 to 2021, 65.2% (*n* = 105/161) of the manuscripts had women first authors (*p* = 0.023).

**Figure 2:**
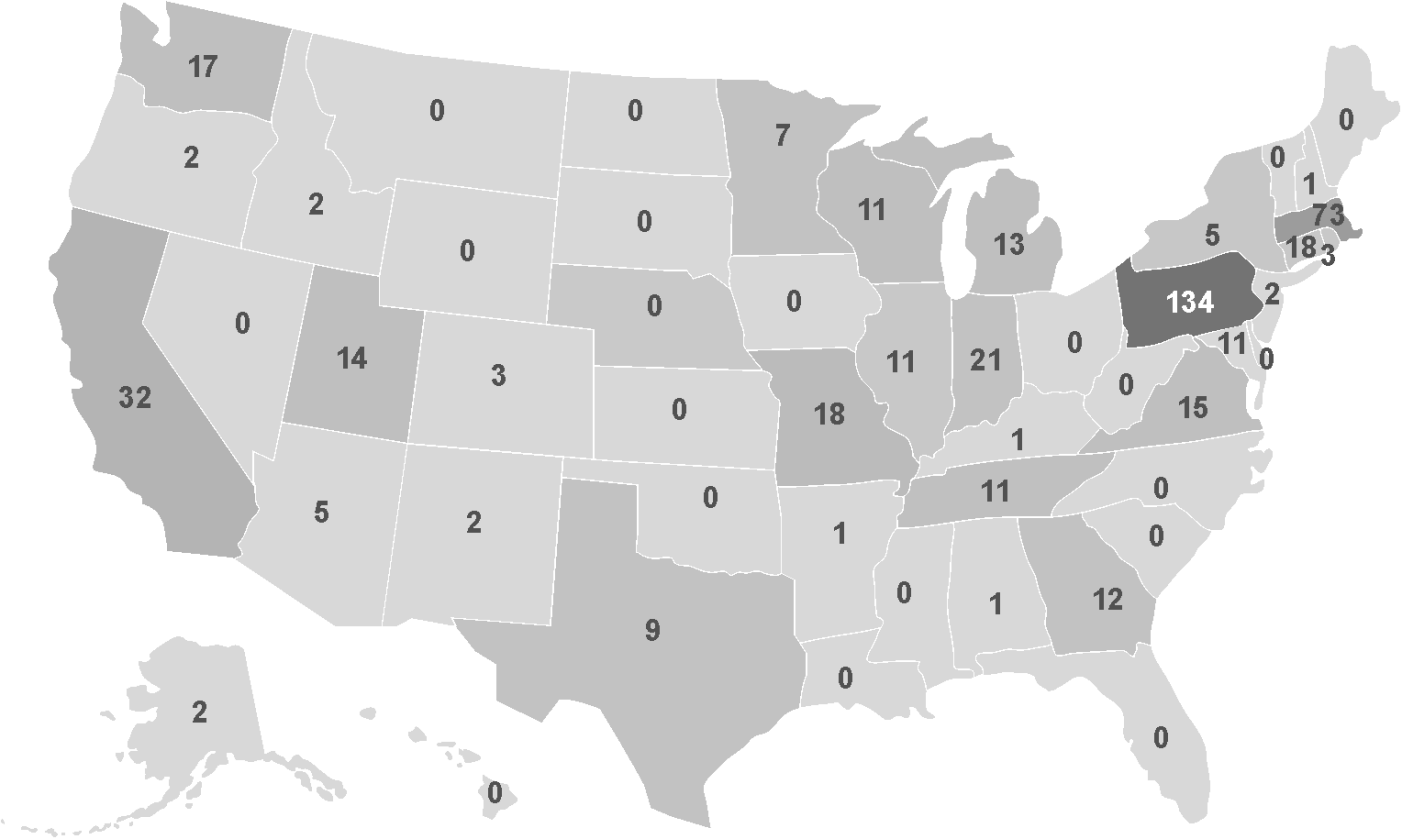
Papers published by state: this map of the United States depicts the number of articles published with the institutional affiliation of the first or senior author in that state.

Of the 225 articles that listed a professional role on their masthead, 83.9% (*n* = 188) of the first authors were found to be physicians. In these 225 articles, most non-physician first authors were women (83.8%, 31/37, *p* = 0.001) (Table 1). The vast majority of authors, 83.8% (*n* = 253) were in the United States, while 16.2% (*n* = 49) were in other countries, with women more often represented in the U.S. (*p* = 0.001) (Table 2 and Figure 2). First authors were most commonly from Pennsylvania (19.8%, *n* = 50), Massachusetts (13.4%, *n* = 34), New York (8.3%, *n* = 21), or California (7.1%, *n* = 18).

**Table 1:** Author characteristics by gender.

Variable	Women	Men	*P* value
First Author	168/302 (55.6)	134/302 (44.4)	
Professional role *n* = 225			0.001
Physician (*n* = 188)	93/124 (75.0)	95/101 (94.1)	
PhD (*n* = 16)	15/124 (12.1)	1/101 (1.0)	
Nurse (*n* = 7)	7/124 (5.7)	0/101 (0.0)	
Other (*n* = 14)	9/124 (7.3)	5/101 (5.0)	
United States	151/168 (89.9)	102/134 (76.1)	0.001
Co-First author	9/168 (5.4)	7/134 (5.2)	0.959
Woman senior author	69/165 (41.8)	33/118 (28.0)	0.017
Funded work^a^	131/168 (78.0)	109/134 (81.3)	0.472
NIH	70/131 (53.4)	37/109 (27.9)	0.002
CDC	10/131 (7.6)	4/109 (3.7)	0.192
AHRQ	16/131 (12.2)	13/109 (11.9)	0.946
Foundation	32/131 (24.4)	42/109 (38.5)	0.018
Institutional	43/131 (32.8)	39 (35.8)	0.631
Senior Author	102/283 (36.0)	181/283 (64.0)	
Professional role *n* = 210			<0.001
Physician	46/72 (63.9)	137/138 (99.3)	
PhD	12/72 (16.7)	1/138 (0.7)	
Nurse	14/72 (19.4)	0/138 (0.0)	
United States	91/102 (89.2)	154/181 (85.1)	0.328
Co-Senior Author	5/102 (4.9)	4/181 (2.2)	0.213
Funded work^a^	78/102 (76.5)	145/181 (80.1)	0.472
NIH	48/78 (61.5)	59/145 (40.7)	0.003
CDC	12/78 (15.4)	2/145 (1.4)	<0.001
AHRQ	1/78 (1.3)	28/145 (19.3)	<0.001
Foundation	8/78 (10.3)	49/145 (33.8)	<0.001
Institutional	17/78 (21.8)	64 /145 (44.1)	0.001
Corresponding Author	139/284 (48.9)	145/284 (51.0)	

Results are presented as counts with percentages in parentheses and were compared using chi-squared analysis.

NIH, National Institutes of Health; CDC, Center for Disease Control; AHRQ, Agency for Healthcare Research and Quality.

^a^ Funding is per manuscript, not per author.

**Table 2:** First and senior authors by country.

Country	First author	Senior author
	*N* = 302	*N* = 283
United States	253 (83.8)	245 (86.6)
Canada	29 (9.6)	26 (9.2)
France	1 (0.3)	4 (1.4)
Switzerland	4 (1.3)	1 (0.4)
United Kingdom	2 (0.7)	3 (1.1)
Australia	2 (0.7)	1 (0.4)
Belgium	3 (1.0)	0 (0.0)
Netherlands	2 (0.7)	1 (0.4)
Singapore	2 (0.7)	0 (0.0)
Spain	2 (0.7)	0 (0.0)
China	0 (0.0)	1 (0.4)
Germany	0 (0.0)	1 (0.4)
Malaysia	1 (0.3)	0 (0.0)
South Africa	1 (0.3)	0 (0.0)

### Senior authorship

Of the 283 senior authors, 36.0% (*n* = 102) were found to be women and represented 119 unique individuals, of whom 36.1% (*n* = 43) were found to be women. There were 34.1% (*n* = 15) senior women authors listed between 2002 and 2011 and 36.4% (*n* = 87) between 2012 and 2021, with no significant change over time (*p* = 0.708); this persisted when isolating unique senior authors. Of the 209 articles with senior authors that listed a professional role on their masthead, 64.7% (*n* = 183) were found to be physicians and 25.1% (*n* = 46) were found to be women. Nearly all non-physician authors (PhDs, Nurses, etc.) were women (Table 1). The majority, 86.6% (*n* = 245) of senior authors, were from the United States, with the most common states represented being Pennsylvania (34.3%), Massachusetts (15.9%), and California (5.7%).

A higher proportion of manuscripts with women senior authors had a women first author, 67.7% (*n* = 69) vs., 32.4% (*n* = 330, *p* = 0.017), and manuscripts with a women senior author were more likely to have a woman first author: OR = 1.9 (95%, CI 1.1, 3.1, *p* = 0.017). The majority of articles (284/302) had a corresponding author listed, and if listed, that author is a woman 48.9% (*n* = 139) of the time (Table 1).

### Journals and funding

Of the 254 manuscripts that had an impact factor in the year of publication, there was no difference in median impact factor based on gender of the first author (women 3.5 [IQR: 2.8, 7.0] vs. men 3.7 [IQR: 2.8, 7.6], *p* = 0.444) or senior author (women 3.6 [IQR: 2.8, 6.6] vs. men 3.5 [IQR: 2.8, 7.4], *p* = 0.419). The majority of articles, 43.2% (*n* = 131) were published in a single journal, *Pediatric Critical Care Medicine (PCCM),* with *CCM* publishing 11.9% (*n* = 36) of the articles, and the remainder of the articles spread out over an additional 61 journals (Supplementary Table S1). Impact factors varied over time; *PCCM* was not indexed until 2008.

Funding was disclosed in 240/302 articles and did not differ by gender (*p* = 0.472), with multiple funding sources being common (Table 1). When examining specific funding types, there were some differences by gender. Women first authors and women senior authors had a higher proportion of manuscripts whose work was funded by the National Institute of Health [70/131 (53.4%) vs. 37/109 (27.9%), *p* = 0.002, and 48/78 (61.5%) vs. 59/145 (40.7%), *p* = 0.003, respectively]. Women first authors and women senior authors published fewer manuscripts funded by foundations [32/131 (24.4%) vs. 42/109 (38.5%), *p* = 0.018, and 8/78 (10.3%) vs. 49/145 (33.8%), *p* < 0.001, respectively]. Women senior authors were more likely to have Center for Disease Control funding [12/78 (15.4%) vs. 2/145 (1.4%), *p* < 0.001] but less likely to have AHRQ [1/78 (1.3%) vs. 28/145 (19.3%), *p* < 0.001] and institutional funding [17/78 (21.8%) vs. 64/145 (44.1%), *p* = 0.001].

## Discussion

Our investigation of gender and authorship in PALISI publications demonstrates an increase in the proportion of women first authors over time (to 65% between 2017 and 2021), which has outpaced the increase in the number of women physicians in pediatric critical care (50% in 2021). However, the proportion of women senior authors has stagnat has been stagnant, reflecting major opportunities for the advancement of women as senior researchers and leaders. PALISI has a history of mentoring and encouraging junior investigators (9). When PALISI was founded in 2002, 35.7% of those who passed their pediatric critical care boards were women, and 41.2% of senior fellows were women (2). In total, 37.1% of women first authors between 2002 and 2012 are consistent with this demographic. With 64.8% of the first authors of PALISI publications from 2017 through 2021 being women, the gender of the first author position has outpaced the increase of women in pediatric critical care medicine (49.6% in 2021) (1). Utilizing pediatric critical care medicine physicians as the comparison group has some limitations, as only two-thirds of the first authors were physicians. Since virtually all of the non-physician first authors were women, this may account for the slight increase in first authorship compared to the 2020 data for practicing pediatric critical care physicians. However, the first authors being predominantly women in the most recent period is very different from other recently published similar studies that examined the work product of the Canadian Critical Care Trials Group (11), high-impact critical care publications (12), all papers published in *CCM* (3), and pediatric critical care randomized control trials (13). The first two articles showed no difference in women first authorship over time and the latter two showed an increase in women first authors over time, but only to 25% in 2010–2021 and 39% in 2015–2018 respectively. This still lags far behind the 59% women authorship in PALISI from 2012–2021. This significantly greater proportion of women first authors in PALISI's work product shows the dedication and intentionality of the network to all junior investigators (9).

While women first authorship has increased over time, the same is not true for senior authorship, as the percentage of women senior authors has stagnated over time. We know that women often do not advance into senior leadership positions (3, 6, 14–16). They are underrepresented in critical care societies (17), critical care task forces (18), and journal editors (3), and in general, their numbers decline at each step of the academic ladder (6). While it is inspiring that PALISI has successfully supported emerging women scientists, as evidenced by the rising proportion of women first authors, the network now needs to find a way to close the gap at the senior level. Our data show that one-third of the senior women authors are non-physicians, while virtually none of the senior male authors are non-physicians. On the one hand, this shows the diversity of non-physician women scientists in interprofessional roles, but it also underscores that women physicians may not be ascending the academic ladder. It is unclear how gender affects funding, as seen through PALISI publications. Women had more NIH and CDC funding but less foundation and AHRQ funding. The implications of the differences in funding, while statistically significant, are unclear.

Our findings on PALISI publications are consistent with other data showing that women senior authorship (12, 19) confers an effect on increasing woman first authorship. With the high frequency of woman first authorship and its rise in the last 10 years, it surprised us that woman senior authorship confers such an advantage. This may be due to unconscious and implicit biases like the invisibility of mid-career women as suggested by *Lewiss* et al. and traditional gender norms as suggested by *Chadwick* et al. (7, 16). This is not all due to a single senior author with many publications and large trials, as there were 122 unique senior authors among the 303 publications and no single individual dominated the field.

With nearly half of PALISI's work published in a single journal and 55.4% of the articles published in the Society for Critical Care Medicine family of journals, it is important to look at the gender of the editors-in-chief and editorial boards of these journals. There have been no woman editors-in-chief since their inception. The editorial board at *PCCM* is approximately 27% women, that at *CCM* is 29% women, and the editorial board at *Critical Care Explorations* is 50% women (3). In all three of these journals, reviewers are not blinded to the authors and are potentially aware of the author's gender. Double blinding of the review process, which is widely supported by the research community, has long been discussed as a method to reduce multiple biases, including gender bias (19–21). It is important to note that this may not increase women authorship, as shown by Williams *et al*. when they looked at the gender of all reviewers and authors over a 2-year period in *The Journal of Pediatrics*. They found no correlations with the gender of either authors or reviewers regarding acceptance for publication, but did find that fewer women were offered the opportunity to serve as reviewers and, when offered the opportunity to review a manuscript, were less likely to accept it, thereby exacerbating the gender parity of reviewers (5).

As we studied articles published through 2021, it is unclear whether the COVID-19 pandemic will affect PALISI productivity and, if there is an effect, whether it will disproportionately influence authorship by women. There is copious evidence that women were less academically productive in the early stages of the pandemic and that COVID-19 could set back women's progress in the workforce by as much as two decades (22–25), although there is scholarship supporting methodology to overcome these inequities (26).

PALISI's founder and executive chair from 2002 to 2014, Dr. Adrienne Randolph, and its second executive chair from 2014 to 2017, Dr. Ann Thompson, are the epitome of strong, established, senior women in the field who very much lead by example. Since 2017, there have been no women serving as executive chairs. PALISI's current executive committee is 50% (*n* = 5) women, and its scientific committee is 80% (*n* = 4) women (27). While PALISI's track record for women first authorship is impressive, there is still work to be done to promote senior women authors. Individuals, institutions, research networks, professional societies, and their journals all need to work in concert to keep women climbing the academic ladder, while individual women need to be proactive and thoughtful about speaking and reviewing (5, 18, 28). Members of PALISI (as with any research network) need to be conscientious in their voting for committee members, their invitations to speakers, and their own scholarship in terms of whom they mentor and sponsor. Institutions composed of academic medical centers, hospitals, healthcare organizations, and practices need to use a scientific approach that includes metrics, analysis, and transparent reporting of outcomes to demonstrate impact (29). Professional societies need to diversify their planning committees, speakers, and chairs, and then track and publish their changes (3). Additionally, virtual meeting attendance, childcare, and nursing facilities can all increase women's ability to participate in all events (28). Journals have an obligation to tackle both conscious (explicit) and unconscious (implicit) bias (30). Together, in an effort to reduce gender bias, we are likely to also reduce other biases, hopefully leading to a more robust representation of all in pediatric critical care medicine.

This study has several limitations. It is possible, despite our efforts, that we may have unintentionally omitted some articles that were work products of PALISI from the analysis. We do not know the gender makeup of PALISI, as the network has never collected this type of data. While we use the gender makeup of currently practicing pediatric intensivists to estimate how many women would be eligible to write articles, there are many non-intensivists who attend and participate in PALISI. Our data showed 62.3% of first authors were physicians which is consistent with unofficial membership data as per personal communication with Dr. Neal Thomas Geographic data may show initiatives by academic institutions and have no association with PALISI. We do not have additional demographic data on individuals beyond gender. We did not include co-first and co-senior authors in our analysis. Despite these limitations, this study accurately reflects the work product of the PALISI network as a whole. Future work with a potential prospective collection of author gender, with additional demographic data, may help us ensure future comprehensive assessments of representation in pediatric critical care research.

## Conclusions

PALISI, as a network, has had a robust publication record in its first 20 years. Authorship by women in the first author position has increased over time, currently exceeding both the number of women practicing pediatric critical care and the number of women first authors in critical care publications as a whole. Women senior authorship has been stagnant since the network's inception. A multifactorial approach by individuals, institutions, networks, and journals is needed to increase senior women authorship.

## Data Availability

The raw data supporting the conclusions of this article will be made available by the authors, without undue reservation.
