# Supplementary material for: Gender and authorship of publications from Pediatric Acute Lung Injury and Sepsis Investigators (PALISI)
Source: Front Pediatr. 2023 Dec 19;11:1318690. doi: 10.3389/fped.2023.1318690 (PMC10758432; doi:10.3389/fped.2023.1318690)
Supplement: Supplementary file 1 [file Table1.docx]

Gender and Authorship of Publications from Pediatric Acute Lung Injury and Sepsis Investigators (PALISI)

Asumthia S. Jeyapalan DO MHA^1^, Stephanie R. Brown MBBS, MS^2^, Mary G. Gaspers MD MPH^3^, Brittany Haliani^4^, Sapna R. Kudchadkar MD PhD FCCM^5^, Courtney M. Rowan MD, MScr^6^, Shira J. Gertz MD*^7^, for the Pediatric Acute Lung Injury and Sepsis Investigators (PALISI) Network

**Affiliations**

1. University of Miami, Division of Critical Care Medicine, Department of Pediatrics, Miami, FL, USA
2. University of Oklahoma, Division of Pediatric Critical Care, Oklahoma City, OK, USA
3. University of Arizona, Department of Pediatrics and Banner Children's at Diamond Children's Medical Center, Tucson, AZ, USA
4. Cooperman Barnabas Medical Center, Medical Librarian, Livingston, NJ, USA
5. Johns Hopkins University School of Medicine, Department of Anesthesiology and Critical Care Medicine, Baltimore, MD, USA
6. Indiana University School of Medicine and Riley Hospital for Children at IU Health, Division of Critical Care, Department of Pediatrics, Indianapolis, Indiana, USA
7. Cooperman Barnabas Medical Center, Division of Pediatric Critical Care, Department of Pediatrics, Livingston, NJ, USA

*** Correspondence:**

Shira J. Gertz, MD

shira.gertz@rwjbh.org

Table of Contents: Pages

Appendix 1: Complete Bibliography 3 - 20

Supplemental Table 1: Journals of PALISI Publications 21 - 22

**Appendix 1: Bibliography of works included in analysis**

1. Randolph AG, Wypij D, Venkataraman ST, et al.: Effect of mechanical ventilator weaning protocols on respiratory outcomes in infants and children: a randomized controlled trial. *Jama* 2002; 288:2561-2568

2. Randolph AG, Meert KL, O'Neil ME, et al.: The feasibility of conducting clinical trials in infants and children with acute respiratory failure. *Am J Respir Crit Care Med* 2003; 167:1334-1340

3. Allen UD: Factors influencing predisposition to sepsis in children with cancers and acquired immunodeficiencies unrelated to human immunodeficiency virus infection. *Pediatr Crit Care Med* 2005; 6:S80-86

4. Brilli RJ, Goldstein B: Pediatric sepsis definitions: past, present, and future. *Pediatr Crit Care Med* 2005; 6:S6-8

5. Carcillo JA: Reducing the global burden of sepsis in infants and children: a clinical practice research agenda. *Pediatr Crit Care Med* 2005; 6:S157-164

6. Curley MA, Hibberd PL, Fineman LD, et al.: Effect of prone positioning on clinical outcomes in children with acute lung injury: a randomized controlled trial. *Jama* 2005; 294:229-237

7. Curley MA, Zimmerman JJ: Alternative outcome measures for pediatric clinical sepsis trials. *Pediatr Crit Care Med* 2005; 6:S150-156

8. Dahmer MK, Randolph A, Vitali S, et al.: Genetic polymorphisms in sepsis. *Pediatr Crit Care Med* 2005; 6:S61-73

9. Escobar GJ: What have we learned from observational studies on neonatal sepsis? *Pediatr Crit Care Med* 2005; 6:S138-145

10. Fischer JE: Physicians' ability to diagnose sepsis in newborns and critically ill children. *Pediatr Crit Care Med* 2005; 6:S120-125

11. Goldstein B, Giroir B, Randolph A, et al.: International pediatric sepsis consensus conference: definitions for sepsis and organ dysfunction in pediatrics. *Pediatr Crit Care Med* 2005; 6:2-8

12. Haque KN: Definitions of bloodstream infection in the newborn. *Pediatr Crit Care Med* 2005; 6:S45-49

13. Hatherill M: Sepsis predisposition in children with human immunodeficiency virus. *Pediatr Crit Care Med* 2005; 6:S92-98

14. Hazelzet JA: Diagnosing meningococcemia as a cause of sepsis. *Pediatr Crit Care Med* 2005; 6:S50-54

15. Lacroix J, Cotting J: Severity of illness and organ dysfunction scoring in children. *Pediatr Crit Care Med* 2005; 6:S126-134

16. Langley JM: Defining urinary tract infection in the critically ill child. *Pediatr Crit Care Med* 2005; 6:S25-29

17. Langley JM, Bradley JS: Defining pneumonia in critically ill infants and children. *Pediatr Crit Care Med* 2005; 6:S9-S13

18. Opal SM: Concept of PIRO as a new conceptual framework to understand sepsis. *Pediatr Crit Care Med* 2005; 6:S55-60

19. Orange JS: Congenital immunodeficiencies and sepsis. *Pediatr Crit Care Med* 2005; 6:S99-S107

20. Overturf GD: Defining bacterial meningitis and other infections of the central nervous system. *Pediatr Crit Care Med* 2005; 6:S14-18

21. Priebe GP: Sepsis in the immunocompromised child: the least studied with the most to gain. *Pediatr Crit Care Med* 2005; 6:S78-79

22. Randolph AG, Brun-Buisson C, Goldmann D: Identification of central venous catheter-related infections in infants and children. *Pediatr Crit Care Med* 2005; 6:S19-24

23. Randolph AG, Forbes PW, Gedeit RG, et al.: Cumulative fluid intake minus output is not associated with ventilator weaning duration or extubation outcomes in children. *Pediatr Crit Care Med* 2005; 6:642-647

24. See LL: Bloodstream infection in children. *Pediatr Crit Care Med* 2005; 6:S42-44

25. Sheridan RL: Sepsis in pediatric burn patients. *Pediatr Crit Care Med* 2005; 6:S112-119

26. Tamburro R: Pediatric cancer patients in clinical trials of sepsis: factors that predispose to sepsis and stratify outcome. *Pediatr Crit Care Med* 2005; 6:S87-91

27. Tarnow-Mordi WO: What have we learned about randomized, controlled trials in neonatal sepsis? *Pediatr Crit Care Med* 2005; 6:S146-149

28. Tarnow-Mordi WO: What is the role of neonatal organ dysfunction and illness severity scores in therapeutic studies in sepsis? *Pediatr Crit Care Med* 2005; 6:S135-137

29. Thompson AE, Marshall JC, Opal SM: Intraabdominal infections in infants and children: descriptions and definitions. *Pediatr Crit Care Med* 2005; 6:S30-35

30. Upperman JS, Sheridan RL: Pediatric trauma susceptibility to sepsis. *Pediatr Crit Care Med* 2005; 6:S108-111

31. Upperman JS, Sheridan RL, Marshall J: Pediatric surgical site and soft tissue infections. *Pediatr Crit Care Med* 2005; 6:S36-41

32. Vitali SH, Randolph AG: Assessing the quality of case-control association studies on the genetic basis of sepsis. *Pediatr Crit Care Med* 2005; 6:S74-77

33. Watson RS, Carcillo JA: Scope and epidemiology of pediatric sepsis. *Pediatr Crit Care Med* 2005; 6:S3-5

34. Willson DF, Thomas NJ, Markovitz BP, et al.: Effect of exogenous surfactant (calfactant) in pediatric acute lung injury: a randomized controlled trial. *Jama* 2005; 293:470-476

35. Curley MA, Arnold JH, Thompson JE, et al.: Clinical trial design--effect of prone positioning on clinical outcomes in infants and children with acute respiratory distress syndrome. *J Crit Care* 2006; 21:23-32; discussion 32-27

36. Fineman LD, LaBrecque MA, Shih MC, et al.: Prone positioning can be safely performed in critically ill infants and children. *Pediatr Crit Care Med* 2006; 7:413-422

37. Lacroix J, Hébert PC, Hutchison JS, et al.: Transfusion strategies for patients in pediatric intensive care units. *N Engl J Med* 2007; 356:1609-1619

38. Bateman ST, Lacroix J, Boven K, et al.: Anemia, blood loss, and blood transfusions in North American children in the intensive care unit. *Am J Respir Crit Care Med* 2008; 178:26-33

39. Hirshberg E, Lacroix J, Sward K, et al.: Blood glucose control in critically ill adults and children: a survey on stated practice. *Chest* 2008; 133:1328-1335

40. Morris AH, Orme J, Rocha BH, et al.: An electronic protocol for translation of research results to clinical practice: a preliminary report. *J Diabetes Sci Technol* 2008; 2:802-808

41. Sward K, Orme J, Jr., Sorenson D, et al.: Reasons for declining computerized insulin protocol recommendations: application of a framework. *J Biomed Inform* 2008; 41:488-497

42. Tamburro RF, Thomas NJ, Pon S, et al.: Post hoc analysis of calfactant use in immunocompromised children with acute lung injury: Impact and feasibility of further clinical trials. *Pediatr Crit Care Med* 2008; 9:459-464

43. Thompson BT, Orme JF, Zheng H, et al.: Multicenter validation of a computer-based clinical decision support tool for glucose control in adult and pediatric intensive care units. *J Diabetes Sci Technol* 2008; 2:357-368

44. Gauvin F, Lacroix J: Should we avoid transfusion in the pediatric intensive care unit? *Pediatr Crit Care Med* 2009; 10:400-401

45. Karam O, Tucci M, Toledano BJ, et al.: Length of storage and in vitro immunomodulation induced by prestorage leukoreduced red blood cells. *Transfusion* 2009; 49:2326-2334

46. Lacroix J, Trottier H, Tucci M: [Improving use of red-cell transfusion in pediatric intensive care units]. *Med Sci (Paris)* 2009; 25:963-966

47. Fowler RA, Webb SA, Rowan KM, et al.: Early observational research and registries during the 2009-2010 influenza A pandemic. *Crit Care Med* 2010; 38:e120-132

48. Gauvin F, Spinella PC, Lacroix J, et al.: Association between length of storage of transfused red blood cells and multiple organ dysfunction syndrome in pediatric intensive care patients. *Transfusion* 2010; 50:1902-1913

49. In FGHNC: InFACT: a global critical care research response to H1N1. *Lancet* 2010; 375:11-13

50. Karam O, Tucci M, Bateman ST, et al.: Association between length of storage of red blood cell units and outcome of critically ill children: a prospective observational study. *Crit Care* 2010; 14:R57

51. Rouette J, Trottier H, Ducruet T, et al.: Red blood cell transfusion threshold in postsurgical pediatric intensive care patients: a randomized clinical trial. *Ann Surg* 2010; 251:421-427

52. Santschi M, Jouvet P, Leclerc F, et al.: Acute lung injury in children: therapeutic practice and feasibility of international clinical trials. *Pediatric critical care medicine : a journal of the Society of Critical Care Medicine and the World Federation of Pediatric Intensive and Critical Care Societies* 2010; 11:681-689

53. Spinella PC, Dressler A, Tucci M, et al.: Survey of transfusion policies at US and Canadian children's hospitals in 2008 and 2009. *Transfusion* 2010; 50:2328-2335

54. Thomas NJ, Shaffer ML, Willson DF, et al.: Defining acute lung disease in children with the oxygenation saturation index. *Pediatric Critical Care Medicine* 2010; 11:12-17

55. Willems A, Harrington K, Lacroix J, et al.: Comparison of two red-cell transfusion strategies after pediatric cardiac surgery: a subgroup analysis. *Crit Care Med* 2010; 38:649-656

56. Cholette JM, Rubenstein JS, Alfieris GM, et al.: Children with single-ventricle physiology do not benefit from higher hemoglobin levels post cavopulmonary connection: results of a prospective, randomized, controlled trial of a restrictive versus liberal red-cell transfusion strategy. *Pediatr Crit Care Med* 2011; 12:39-45

57. Faustino EV, Patel S, Thiagarajan RR, et al.: Survey of pharmacologic thromboprophylaxis in critically ill children. *Crit Care Med* 2011; 39:1773-1778

58. Karam O, Tucci M, Ducruet T, et al.: Red blood cell transfusion thresholds in pediatric patients with sepsis. *Pediatr Crit Care Med* 2011; 12:512-518

59. Lacroix J, Tucci M: [Clinical impact of length of storage before red blood cell transfusion]. *Transfus Clin Biol* 2011; 18:97-105

60. McArthur J, Pettersen G, Jouvet P, et al.: The care of critically ill children after hematopoietic SCT: a North American survey. *Bone Marrow Transplant* 2011; 46:227-231

61. Randolph AG, Vaughn F, Sullivan R, et al.: Critically ill children during the 2009-2010 influenza pandemic in the United States. *Pediatrics* 2011; 128:e1450-1458

62. Cholette JM, Henrichs KF, Alfieris GM, et al.: Washing red blood cells and platelets transfused in cardiac surgery reduces postoperative inflammation and number of transfusions: results of a prospective, randomized, controlled clinical trial. *Pediatr Crit Care Med* 2012; 13:290-299

63. Grant MJ, Scoppettuolo LA, Wypij D, et al.: Prospective evaluation of sedation-related adverse events in pediatric patients ventilated for acute respiratory failure. *Crit Care Med* 2012; 40:1317-1323

64. Khemani RG, Thomas NJ, Venkatachalam V, et al.: Comparison of SpO2 to PaO2 based markers of lung disease severity for children with acute lung injury. *Crit Care Med* 2012; 40:1309-1316

65. Muszynski J, Nateri J, Nicol K, et al.: Immunosuppressive effects of red blood cells on monocytes are related to both storage time and storage solution. *Transfusion* 2012; 52:794-802

66. Nishisaki A, Ferry S, Colborn S, et al.: Characterization of tracheal intubation process of care and safety outcomes in a tertiary pediatric intensive care unit. *Pediatr Crit Care Med* 2012; 13:e5-10

67. Rice TW, Rubinson L, Uyeki TM, et al.: Critical illness from 2009 pandemic influenza A virus and bacterial coinfection in the United States. *Crit Care Med* 2012; 40:1487-1498

68. Thomas NJ, Guardia CG, Moya FR, et al.: A pilot, randomized, controlled clinical trial of lucinactant, a peptide-containing synthetic surfactant, in infants with acute hypoxemic respiratory failure. *Pediatr Crit Care Med* 2012; 13:646-653

69. Trottier H, Buteau C, Robitaille N, et al.: Transfusion-related Epstein-Barr virus infection among stem cell transplant recipients: a retrospective cohort study in children. *Transfusion* 2012; 52:2653-2663

70. Valentine SL, Bateman ST: Identifying factors to minimize phlebotomy-induced blood loss in the pediatric intensive care unit. *Pediatr Crit Care Med* 2012; 13:22-27

71. Valentine SL, Sapru A, Higgerson RA, et al.: Fluid balance in critically ill children with acute lung injury. *Crit Care Med* 2012; 40:2883-2889

72. Cholette JM, Powers KS, Alfieris GM, et al.: Transfusion of cell saver salvaged blood in neonates and infants undergoing open heart surgery significantly reduces RBC and coagulant product transfusions and donor exposures: results of a prospective, randomized, clinical trial. *Pediatr Crit Care Med* 2013; 14:137-147

73. Duncan CN, Lehmann LE, Cheifetz IM, et al.: No Title. *Pediatric Critical Care Medicine* 2013; 14:261-267

74. Faustino EV, Spinella PC, Li S, et al.: Incidence and acute complications of asymptomatic central venous catheter-related deep venous thrombosis in critically ill children. *J Pediatr* 2013; 162:387-391

75. Grant MJ, Balas MC, Curley MA, et al.: Defining sedation-related adverse events in the pediatric intensive care unit. *Heart Lung* 2013; 42:171-176

76. Hall MW, Geyer SM, Guo CY, et al.: Innate immune function and mortality in critically ill children with influenza: a multicenter study. *Crit Care Med* 2013; 41:224-236

77. Hirshberg EL, Sward KA, Faustino EV, et al.: Clinical equipoise regarding glycemic control: a survey of pediatric intensivist perceptions. *Pediatr Crit Care Med* 2013; 14:123-129

78. Nishisaki A, Turner DA, Brown CA, 3rd, et al.: A National Emergency Airway Registry for children: landscape of tracheal intubation in 15 PICUs. *Crit Care Med* 2013; 41:874-885

79. Sanders RC, Jr., Giuliano JS, Jr., Sullivan JE, et al.: Level of trainee and tracheal intubation outcomes. *Pediatrics* 2013; 131:e821-828

80. Santschi M, Randolph AG, Rimensberger PC, et al.: Mechanical ventilation strategies in children with acute lung injury: a survey on stated practice pattern*. *Pediatr Crit Care Med* 2013; 14:e332-337

81. Thomas NJ, Jouvet P, Willson D: Acute lung injury in children--kids really aren't just "little adults". *Pediatr Crit Care Med* 2013; 14:429-432

82. Willson DF, Thomas NJ, Tamburro R, et al.: Pediatric calfactant in acute respiratory distress syndrome trial. *Pediatr Crit Care Med* 2013; 14:657-665

83. Aspesberro F, Milewski LS, Brogan TV: Acute central nervous system complications in pediatric hematopoietic stem cell patients. *J Pediatr Intensive Care* 2014; 3:169-181

84. Bennett TD, Spaeder MC, Matos RI, et al.: Existing data analysis in pediatric critical care research. *Front Pediatr* 2014; 2:79

85. Faustino EVS, Hanson S, Spinella PC, et al.: A multinational study of thromboprophylaxis practice in critically ill children. *Critical Care Medicine* 2014; 42:1232-1240

86. Ferdinands JM, Olsho LE, Agan AA, et al.: Effectiveness of influenza vaccine against life-threatening RT-PCR-confirmed influenza illness in US children, 2010-2012. *J Infect Dis* 2014; 210:674-683

87. Fitzgerald JC, Williams D, Laskin BL: Acute kidney injury in pediatric hematopoietic stem cell transplant recipients. *J Pediatr Intensive Care* 2014; 3:159-168

88. Graciano AL, Tamburro R, Thompson AE, et al.: Incidence and associated factors of difficult tracheal intubations in pediatric ICUs: a report from National Emergency Airway Registry for Children: NEAR4KIDS. *Intensive Care Med* 2014; 40:1659-1669

89. Higgerson RA, Olsho LE, Christie LM, et al.: Variability in IRBs regarding parental acceptance of passive consent. *Pediatrics* 2014; 134:e496-503

90. Hume JR, Gupta S, Steiner ME: Historical outcomes of pediatric hematopoietic stem cell transplantation patients requiring critical care. *J Pediatr Intensive Care* 2014; 3:83-90

91. Klein OR, Cooke KR: Idiopathic pneumonia syndrome following hematopoietic stem cell transplantation. *Journal of Pediatric Intensive Care* 2014; 3:147-157

92. Mahadeo KM, Bajwa RPS: Hepatic veno-occlusive disease in children after hematopoietic stem cell transplantation. *J Pediatr Intensive Care* 2014; 3:183-193

93. Moffet JR, Stafford LA, Stokhuyzen AR, et al.: A review of graft versus host disease. *J Pediatr Intensive Care* 2014; 3:103-113

94. Needle J: Ethical challenges with the advancement of hematopoietic stem cell transplant. *J Pediatr Intensive Care* 2014; 3:195-200

95. Nett S, Emeriaud G, Jarvis JD, et al.: Site-level variance for adverse tracheal intubation-associated events across 15 North American PICUs: a report from the national emergency airway registry for children*. *Pediatr Crit Care Med* 2014; 15:306-313

96. Rehder KJ, Cheifetz IM, Markovitz BP, et al.: Survey of in-house coverage by pediatric intensivists: characterization of 24/7 in-hospital pediatric critical care faculty coverage*. *Pediatr Crit Care Med* 2014; 15:97-104

97. Rehder KJ, Cheifetz IM, Willson DF, et al.: Perceptions of 24/7 in-hospital intensivist coverage on pediatric housestaff education. *Pediatrics* 2014; 133:88-95

98. Rowan C, Baloglu O, McArthur J: Non-infectious pulmonary complications of hematopoietic stem cell transplantation. *J Pediatr Intensive Care* 2014; 3:133-146

99. Tamburro RF: Hematopoietic stem cell transplantation in pediatric critical care. *J Pediatr Intensive Care* 2014; 3:81-82

100. Walsh K, Margossian S, Duncan CN: Pediatric hematopoietic cell transplantation. *J Pediatr Intensive Care* 2014; 3:91-101

101. Weiss SL, Fitzgerald JC, Faustino EV, et al.: Understanding the global epidemiology of pediatric critical illness: the power, pitfalls, and practicalities of point prevalence studies. *Pediatric critical care medicine : a journal of the Society of Critical Care Medicine and the World Federation of Pediatric Intensive and Critical Care Societies* 2014; 15:660-666

102. Willson DF, Kirby A, Kicker JS: Respiratory secretion analyses in the evaluation of ventilator-associated pneumonia: a survey of current practice in pediatric critical care. *Pediatr Crit Care Med* 2014; 15:715-719

103. Zinter MS, Levy ER, Gertz SJ: A review of pathogens causing lower respiratory tract infection in the pediatric hematopoietic stem cell transplant recipient. *J Pediatr Intensive Care* 2014; 3:115-132

104. Bembea MM, Jouvet P, Willson D, et al.: Methodology of the Pediatric Acute Lung Injury Consensus Conference. *Pediatr Crit Care Med* 2015; 16:S1-5

105. Curley MA, Wypij D, Watson RS, et al.: Protocolized sedation vs usual care in pediatric patients mechanically ventilated for acute respiratory failure: a randomized clinical trial. *JAMA* 2015; 313:379-389

106. Dalton HJ, Macrae DJ, Pediatric Acute Lung Injury Consensus Conference G: Extracorporeal support in children with pediatric acute respiratory distress syndrome: proceedings from the Pediatric Acute Lung Injury Consensus Conference. *Pediatr Crit Care Med* 2015; 16:S111-117

107. Emeriaud G, Newth CJ, Pediatric Acute Lung Injury Consensus Conference G: Monitoring of children with pediatric acute respiratory distress syndrome: proceedings from the Pediatric Acute Lung Injury Consensus Conference. *Pediatr Crit Care Med* 2015; 16:S86-101

108. Essouri S, Carroll C: Noninvasive support and ventilation for pediatric acute respiratory distress syndrome: proceedings from the Pediatric Acute Lung Injury Consensus Conference. *Pediatr Crit Care Med* 2015; 16:S102-110

109. Flori H, Dahmer MK, Sapru A, et al.: Comorbidities and assessment of severity of pediatric acute respiratory distress syndrome: proceedings from the Pediatric Acute Lung Injury Consensus Conference. *Pediatr Crit Care Med* 2015; 16:S41-50

110. Karam O, Demaret P, Shefler A, et al.: Indications and effects of plasma transfusions in critically ill children. *American Journal of Respiratory and Critical Care Medicine* 2015; 191:1395-1402

111. Khemani RG, Smith LS, Zimmerman JJ, et al.: Pediatric acute respiratory distress syndrome: definition, incidence, and epidemiology: proceedings from the Pediatric Acute Lung Injury Consensus Conference. *Pediatr Crit Care Med* 2015; 16:S23-40

112. Mazine A, Rached-D'Astous S, Ducruet T, et al.: Blood Transfusions After Pediatric Cardiac Operations: A North American Multicenter Prospective Study. *Ann Thorac Surg* 2015; 100:671-677

113. Muszynski JA, Frazier E, Nofziger R, et al.: Red blood cell transfusion and immune function in critically ill children: a prospective observational study. *Transfusion* 2015; 55:766-774

114. Pediatric Acute Lung Injury Consensus Conference G: Pediatric acute respiratory distress syndrome: consensus recommendations from the Pediatric Acute Lung Injury Consensus Conference. *Pediatric Critical Care Medicine* 2015; 16:428-439

115. Quasney MW, Lopez-Fernandez YM, Santschi M, et al.: The outcomes of children with pediatric acute respiratory distress syndrome: proceedings from the Pediatric Acute Lung Injury Consensus Conference. *Pediatr Crit Care Med* 2015; 16:S118-131

116. Rehder KJ, Giuliano JS, Jr., Napolitano N, et al.: Increased Occurrence of Tracheal Intubation-Associated Events During Nights and Weekends in the PICU. *Crit Care Med* 2015; 43:2668-2674

117. Rimensberger PC, Cheifetz IM, Pediatric Acute Lung Injury Consensus Conference G: Ventilatory support in children with pediatric acute respiratory distress syndrome: proceedings from the Pediatric Acute Lung Injury Consensus Conference. *Pediatr Crit Care Med* 2015; 16:S51-60

118. Rodriguez Nunez A, Fonte M, Faustino EV, et al.: [Thromboprophylaxis in critically ill children in Spain and Portugal]. *An Pediatr (Barc)* 2015; 82:144-151

119. Sapru A, Flori H, Quasney MW, et al.: Pathobiology of acute respiratory distress syndrome. *Pediatr Crit Care Med* 2015; 16:S6-22

120. Tamburro RF, Kneyber MCJ, Pediatric Acute Lung Injury Consensus Conference G: Pulmonary specific ancillary treatment for pediatric acute respiratory distress syndrome: proceedings from the Pediatric Acute Lung Injury Consensus Conference. *Pediatric critical care medicine : a journal of the Society of Critical Care Medicine and the World Federation of Pediatric Intensive and Critical Care Societies* 2015; 16:S61-72

121. Tarquinio KM, Howell JD, Montgomery V, et al.: Current medication practice and tracheal intubation safety outcomes from a prospective multicenter observational cohort study. *Pediatr Crit Care Med* 2015; 16:210-218

122. Valentine SL, Nadkarni VM, Curley MA, et al.: Nonpulmonary treatments for pediatric acute respiratory distress syndrome: proceedings from the Pediatric Acute Lung Injury Consensus Conference. *Pediatr Crit Care Med* 2015; 16:S73-85

123. Weiss SL, Fitzgerald JC, Maffei FA, et al.: Discordant identification of pediatric severe sepsis by research and clinical definitions in the SPROUT international point prevalence study for the SPROUT Study Investigators and Pediatric Acute Lung Injury and Sepsis Investigators (PALISI) Network. *Critical Care* 2015; 19

124. Weiss SL, Fitzgerald JC, Pappachan J, et al.: Global epidemiology of pediatric severe sepsis: the sepsis prevalence, outcomes, and therapies study. *American journal of respiratory and critical care medicine* 2015; 191:1147-1157

125. Bateman ST, Borasino S, Asaro LA, et al.: Early High Frequency Oscillatory Ventilation in Pediatric Acute Respiratory Failure: A Propensity Score Analysis. *American Journal of Respiratory & Critical Care Medicine* 2015

126. Best KM, Asaro LA, Franck LS, et al.: Patterns of Sedation Weaning in Critically Ill Children Recovering From Acute Respiratory Failure. *Pediatr Crit Care Med* 2016; 17:19-29

127. Crulli B, Loron G, Nishisaki A, et al.: Safety of paediatric tracheal intubation after non-invasive ventilation failure. *Pediatr Pulmonol* 2016; 51:165-172

128. Fitzgerald JC, Basu RK, Akcan-Arikan A, et al.: Acute Kidney Injury in Pediatric Severe Sepsis: An Independent Risk Factor for Death and New Disability. *Critical Care Medicine* 2016; 44:2241-2250

129. Giuliano JS, Markovitz BP, Brierley J, et al.: Comparison of Pediatric Severe Sepsis Managed in U.S. and European ICUs. *Pediatric Critical Care Medicine* 2016; 17

130. Grant MJ, Schneider JB, Asaro LA, et al.: Dexmedetomidine Use in Critically Ill Children With Acute Respiratory Failure. *Pediatr Crit Care Med* 2016; 17:1131-1141

131. Ishizuka M, Rangarajan V, Sawyer TL, et al.: The Development of Tracheal Intubation Proficiency Outside the Operating Suite During Pediatric Critical Care Medicine Fellowship Training: A Retrospective Cohort Study Using Cumulative Sum Analysis. *Pediatr Crit Care Med* 2016; 17:e309-316

132. Karam O, Demaret P, Duhamel A, et al.: Performance of the PEdiatric Logistic Organ Dysfunction-2 score in critically ill children requiring plasma transfusions. *Annals of Intensive Care* 2016; 6:98-98

133. Lee JH, Turner DA, Kamat P, et al.: The number of tracheal intubation attempts matters! A prospective multi-institutional pediatric observational study. *BMC Pediatr* 2016; 16:58

134. Li S, Rehder KJ, Giuliano JS, Jr., et al.: Development of a Quality Improvement Bundle to Reduce Tracheal Intubation-Associated Events in Pediatric ICUs. *Am J Med Qual* 2016; 31:47-55

135. Onyeama SJ, Hanson SJ, Dasgupta M, et al.: Factors Associated With Continuous Low-Dose Heparin Infusion for Central Venous Catheter Patency in Critically Ill Children Worldwide. *Pediatr Crit Care Med* 2016; 17:e352-361

136. Randolph AG, Agan AA, Flanagan RF, et al.: Optimizing Virus Identification in Critically Ill Children Suspected of Having an Acute Severe Viral Infection. *Pediatr Crit Care Med* 2016; 17:279-286

137. Rowan CM, Gertz SJ, McArthur J, et al.: Invasive Mechanical Ventilation and Mortality in Pediatric Hematopoietic Stem Cell Transplantation: A Multicenter Study. *Pediatric Critical Care Medicine* 2016; 17

138. Sanders RC, Jr., Nett ST, Davis KF, et al.: Family Presence During Pediatric Tracheal Intubations. *JAMA Pediatr* 2016; 170:e154627

139. Shiima Y, Berg RA, Bogner HR, et al.: Cardiac Arrests Associated With Tracheal Intubations in PICUs: A Multicenter Cohort Study. *Crit Care Med* 2016; 44:1675-1682

140. Skeens MA, McArthur J, Cheifetz IM, et al.: High Variability in the Reported Management of Hepatic Veno-Occlusive Disease in Children after Hematopoietic Stem Cell Transplantation. *Biol Blood Marrow Transplant* 2016; 22:1823-1828

141. Ward SL, Gildengorin V, Valentine SL, et al.: Impact of Weight Extremes on Clinical Outcomes in Pediatric Acute Respiratory Distress Syndrome. *Crit Care Med* 2016; 44:2052-2059

142. Yu KO, Randolph AG, Agan AA, et al.: Staphylococcus aureus α-Toxin Response Distinguishes Respiratory Virus-Methicillin-Resistant S. aureus Coinfection in Children. *J Infect Dis* 2016; 214:1638-1646

143. Abu-Sultaneh S, Hobson MJ, Wilson AC, et al.: Practice Variation in the Immediate Postoperative Care of Pediatric Kidney Transplantation: A National Survey. *Transplant Proc* 2017; 49:2060-2064

144. Agus MS, Hirshberg E, Srinivasan V, et al.: Design and rationale of Heart and Lung Failure - Pediatric INsulin Titration Trial (HALF-PINT): A randomized clinical trial of tight glycemic control in hyperglycemic critically ill children. *Contemp Clin Trials* 2017; 53:178-187

145. Agus MS, Wypij D, Hirshberg EL, et al.: Tight Glycemic Control in Critically Ill Children. *N Engl J Med* 2017; 376:729-741

146. Allen EK, Randolph AG, Bhangale T, et al.: SNP-mediated disruption of CTCF binding at the IFITM3 promoter is associated with risk of severe influenza in humans. *Nat Med* 2017; 23:975-983

147. Bajwa RPS, Mahadeo KM, Taragin BH, et al.: Consensus Report by Pediatric Acute Lung Injury and Sepsis Investigators and Pediatric Blood and Marrow Transplantation Consortium Joint Working Committees: Supportive Care Guidelines for Management of Veno-Occlusive Disease in Children and Adolescents, Part 1: Focus on Investigations, Prophylaxis, and Specific Treatment. *Biol Blood Marrow Transplant* 2017; 23:1817-1825

148. Best KM, Wypij D, Asaro LA, et al.: Patient, Process, and System Predictors of Iatrogenic Withdrawal Syndrome in Critically Ill Children. *Crit Care Med* 2017; 45:e7-e15

149. Camazine MN, Karam O, Colvin R, et al.: Outcomes related to the use of frozen plasma or pooled solvent/detergent-treated plasma in critically ill children∗. *Pediatric Critical Care Medicine* 2017; 18:e215-e223

150. Cholette JM, Swartz MF, Rubenstein J, et al.: Outcomes Using a Conservative Versus Liberal Red Blood Cell Transfusion Strategy in Infants Requiring Cardiac Operation. *Ann Thorac Surg* 2017; 103:206-214

151. Davis KF, Napolitano N, Li S, et al.: Promoters and Barriers to Implementation of Tracheal Intubation Airway Safety Bundle: A Mixed-Method Analysis. *Pediatr Crit Care Med* 2017; 18:965-972

152. Demaret P, Karam O, Tucci M, et al.: Anemia at pediatric intensive care unit discharge: prevalence and risk markers. *Ann Intensive Care* 2017; 7:107

153. Faustino EV, Gedeit R, Schwarz AJ, et al.: Accuracy of an Extubation Readiness Test in Predicting Successful Extubation in Children With Acute Respiratory Failure From Lower Respiratory Tract Disease. *Crit Care Med* 2017; 45:94-102

154. Fink EL, Kochanek PM, Tasker RC, et al.: International Survey of Critically Ill Children With Acute Neurologic Insults: The Prevalence of Acute Critical Neurological Disease in Children: A Global Epidemiological Assessment Study. *Pediatr Crit Care Med* 2017; 18:330-342

155. Fiore-Gartland A, Panoskaltsis-Mortari A, Agan AA, et al.: Cytokine Profiles of Severe Influenza Virus-Related Complications in Children. *Front Immunol* 2017; 8:1423

156. Fontela PS, Quach C, Karim ME, et al.: Determinants of Antibiotic Tailoring in Pediatric Intensive Care: A National Survey. *Pediatr Crit Care Med* 2017; 18:e395-e405

157. Grunwell JR, Kamat PP, Miksa M, et al.: Trend and Outcomes of Video Laryngoscope Use Across PICUs. *Pediatr Crit Care Med* 2017; 18:741-749

158. Hartman ME, Saeed MJ, Bennett T, et al.: Readmission and Late Mortality After Critical Illness in Childhood. *Pediatr Crit Care Med* 2017; 18:e112-e121

159. Karam O, Demaret P, Duhamel A, et al.: Factors influencing plasma transfusion practices in paediatric intensive care units around the world. *Vox Sanguinis* 2017

160. Lebet R, Hayakawa J, Chamblee TB, et al.: Maintaining Interrater Agreement of Core Assessment Instruments in a Multisite Randomized Controlled Clinical Trial: The Randomized Evaluation of Sedation Titration for Respiratory Failure (RESTORE) Trial. *Nurs Res* 2017; 66:323-329

161. Lin JC, Spinella PC, Fitzgerald JC, et al.: New or Progressive Multiple Organ Dysfunction Syndrome in Pediatric Severe Sepsis: A Sepsis Phenotype With Higher Morbidity and Mortality. *Pediatr Crit Care Med* 2017; 18:8-16

162. Lindell RB, Gertz SJ, Rowan CM, et al.: High Levels of Morbidity and Mortality Among Pediatric Hematopoietic Cell Transplant Recipients With Severe Sepsis. *Pediatric Critical Care Medicine* 2017:1-1

163. Madsen EC, Levy ER, Madden K, et al.: Mannose-Binding Lectin Levels in Critically Ill Children With Severe Infections. *Pediatr Crit Care Med* 2017; 18:103-111

164. Mahadeo KM, McArthur J, Adams RH, et al.: Consensus Report by the Pediatric Acute Lung Injury and Sepsis Investigators and Pediatric Blood and Marrow Transplant Consortium Joint Working Committees on Supportive Care Guidelines for Management of Veno-Occlusive Disease in Children and Adolescents: Part 2-Focus on Ascites, Fluid and Electrolytes, Renal, and Transfusion Issues. *Biol Blood Marrow Transplant* 2017; 23:2023-2033

165. Muszynski JA, Spinella PC, Cholette JM, et al.: Transfusion-related immunomodulation: review of the literature and implications for pediatric critical illness. *Transfusion* 2017; 57:195-206

166. Natale JE, Lebet R, Joseph JG, et al.: Racial and Ethnic Disparities in Parental Refusal of Consent in a Large, Multisite Pediatric Critical Care Clinical Trial. *J Pediatr* 2017; 184:204-208 e201

167. Parker MM, Nuthall G, Brown C, 3rd, et al.: Relationship Between Adverse Tracheal Intubation Associated Events and PICU Outcomes. *Pediatr Crit Care Med* 2017; 18:310-318

168. Peters MJ, Argent A, Festa M, et al.: The intensive care medicine clinical research agenda in paediatrics. *Intensive Care Med* 2017; 43:1210-1224

169. Randolph AG, Yip WK, Allen EK, et al.: Evaluation of IFITM3 rs12252 Association With Severe Pediatric Influenza Infection. *J Infect Dis* 2017; 216:14-21

170. Roumeliotis N, Ducruet T, Bateman ST, et al.: Determinants of red blood cell transfusion in pediatric trauma patients admitted to the intensive care unit. *Transfusion* 2017; 57:187-194

171. Rowan CM, Smith LS, Loomis A, et al.: Pediatric Acute Respiratory Distress Syndrome in Pediatric Allogeneic Hematopoietic Stem Cell Transplants: A Multicenter Study. *Pediatric Critical Care Medicine* 2017; 18

172. Schneider JB, Sweberg T, Asaro LA, et al.: Sedation Management in Children Supported on Extracorporeal Membrane Oxygenation for Acute Respiratory Failure. *Crit Care Med* 2017; 45:e1001-e1010

173. von Saint André-von Arnim AO, Attebery J, Kortz TB, et al.: Challenges and Priorities for Pediatric Critical Care Clinician-Researchers in Low- and Middle-Income Countries. *Front Pediatr* 2017; 5:277

174. Weiss SL, Asaro LA, Flori HR, et al.: Multiple Organ Dysfunction in Children Mechanically Ventilated for Acute Respiratory Failure. *Pediatr Crit Care Med* 2017; 18:319-329

175. Willson DF, Hoot M, Khemani R, et al.: Pediatric Ventilator-Associated Infections: The Ventilator-Associated INfection Study. *Pediatric Critical Care Medicine* 2017; 18:e24-e34

176. Barbaro RP, Xu Y, Borasino S, et al.: Does Extracorporeal Membrane Oxygenation Improve Survival in Pediatric Acute Respiratory Failure? *American Journal of Respiratory and Critical Care Medicine* 2018:rccm.201709-201893OC

177. Bembea MM, Cheifetz IM, Fortenberry JD, et al.: Recommendations on the Indications for RBC Transfusion for the Critically Ill Child Receiving Support From Extracorporeal Membrane Oxygenation, Ventricular Assist, and Renal Replacement Therapy Devices From the Pediatric Critical Care Transfusion and Anemia Expertise Initiative. *Pediatr Crit Care Med* 2018; 19:S157-s162

178. Bembea MM, Valentine SL, Bateman ST, et al.: The Pediatric Critical Care Transfusion and Anemia Expertise Initiative Consensus Conference Methodology. *Pediatr Crit Care Med* 2018; 19:S93-s97

179. Bigelow AM, Flynn-O'Brien KT, Simpson PM, et al.: Multicenter Review of Current Practices Associated With Venous Thromboembolism Prophylaxis in Pediatric Patients After Trauma. *Pediatr Crit Care Med* 2018; 19:e448-e454

180. Cholette JM, Willems A, Valentine SL, et al.: Recommendations on RBC Transfusion in Infants and Children With Acquired and Congenital Heart Disease From the Pediatric Critical Care Transfusion and Anemia Expertise Initiative. *Pediatr Crit Care Med* 2018; 19:S137-s148

181. Curley MAQ, Gedeit RG, Dodson BL, et al.: Methods in the design and implementation of the Randomized Evaluation of Sedation Titration for Respiratory Failure (RESTORE) clinical trial. *Trials* 2018; 19:687

182. Curley MAQ, Watson RS, Cassidy AM, et al.: Design and rationale of the "Sedation strategy and cognitive outcome after critical illness in early childhood" study. *Contemp Clin Trials* 2018; 72:8-15

183. Dahmer MK, Quasney MW, Sapru A, et al.: Interleukin-1 Receptor Antagonist Is Associated With Pediatric Acute Respiratory Distress Syndrome and Worse Outcomes in Children With Acute Respiratory Failure. *Pediatr Crit Care Med* 2018; 19:930-938

184. Demaret P, Emeriaud G, Hassan NE, et al.: Recommendations on RBC Transfusions in Critically Ill Children With Acute Respiratory Failure From the Pediatric Critical Care Transfusion and Anemia Expertise Initiative. *Pediatr Crit Care Med* 2018; 19:S114-s120

185. Doctor A, Cholette JM, Remy KE, et al.: Recommendations on RBC Transfusion in General Critically Ill Children Based on Hemoglobin and/or Physiologic Thresholds From the Pediatric Critical Care Transfusion and Anemia Expertise Initiative. *Pediatr Crit Care Med* 2018; 19:S98-s113

186. Faustino EVS, Shabanova V, Pinto MG, et al.: Epidemiology of Lower Extremity Deep Venous Thrombosis in Critically Ill Adolescents. *J Pediatr* 2018; 201:176-183 e172

187. Fink EL, von Saint Andre-von Arnim A, Kumar R, et al.: Traumatic Brain Injury and Infectious Encephalopathy in Children From Four Resource-Limited Settings in Africa. *Pediatr Crit Care Med* 2018; 19:649-657

188. Gabrani A, Kojima T, Sanders RC, Jr., et al.: Downward Trend in Pediatric Resident Laryngoscopy Participation in PICUs. *Pediatr Crit Care Med* 2018; 19:e242-e250

189. Gradidge EA, Bakar A, Tellez D, et al.: Effect of Location on Tracheal Intubation Safety in Cardiac Disease-Are Cardiac ICUs Safer? *Pediatr Crit Care Med* 2018; 19:218-227

190. Gradidge EA, Bakar A, Tellez D, et al.: Safety of tracheal intubation in the presence of cardiac disease in paediatric ICUs. *Cardiol Young* 2018; 28:928-937

191. Hassan NE, Reischman DE, Fitzgerald RK, et al.: Hemoglobin Levels Across the Pediatric Critical Care Spectrum: A Point Prevalence Study. *Pediatr Crit Care Med* 2018; 19:e227-e234

192. Hassinger AB, Valentine SL: Self-Reported Management of IV Fluids and Fluid Accumulation in Children With Acute Respiratory Failure. *Pediatr Crit Care Med* 2018; 19:e551-e554

193. Irving SY, Daly B, Verger J, et al.: The association of nutrition status expressed as body mass index z score with outcomes in children with severe sepsis: A secondary analysis from the sepsis prevalence, outcomes, and therapies (SPROUT) study. *Critical Care Medicine* 2018; 46:e1029-e1039

194. Karam O, Russell RT, Stricker P, et al.: Recommendations on RBC Transfusion in Critically Ill Children With Nonlife-Threatening Bleeding or Hemorrhagic Shock From the Pediatric Critical Care Transfusion and Anemia Expertise Initiative. *Pediatr Crit Care Med* 2018; 19:S127-s132

195. Kojima T, Harwayne-Gidansky I, Shenoi AN, et al.: Cricoid Pressure During Induction for Tracheal Intubation in Critically Ill Children: A Report From National Emergency Airway Registry for Children. *Pediatr Crit Care Med* 2018; 19:528-537

196. Kojima T, Laverriere EK, Owen EB, et al.: Clinical Impact of External Laryngeal Manipulation During Laryngoscopy on Tracheal Intubation Success in Critically Ill Children. *Pediatr Crit Care Med* 2018; 19:106-114

197. Langhan ML, Emerson BL, Nett S, et al.: End-Tidal Carbon Dioxide Use for Tracheal Intubation: Analysis From the National Emergency Airway Registry for Children (NEAR4KIDS) Registry. *Pediatr Crit Care Med* 2018; 19:98-105

198. Li S, Hsieh TC, Rehder KJ, et al.: Frequency of Desaturation and Association With Hemodynamic Adverse Events During Tracheal Intubations in PICUs. *Pediatr Crit Care Med* 2018; 19:e41-e50

199. Michelson KN, Reubenson G, Weiss SL, et al.: Site variability in regulatory oversight for an international study of pediatric sepsis. *Pediatric Critical Care Medicine* 2018; 19:e180-e188

200. Muszynski JA, Guzzetta NA, Hall MW, et al.: Recommendations on RBC Transfusions for Critically Ill Children With Nonhemorrhagic Shock From the Pediatric Critical Care Transfusion and Anemia Expertise Initiative. *Pediatr Crit Care Med* 2018; 19:S121-s126

201. Nellis ME, Karam O, Mauer E, et al.: Platelet Transfusion Practices in Critically Ill Children. *Critical Care Medicine* 2018; 46:1309-1317

202. Ovchinsky N, Frazier W, Auletta JJ, et al.: Consensus Report by the Pediatric Acute Lung Injury and Sepsis Investigators and Pediatric Blood and Marrow Transplantation Consortium Joint Working Committees on Supportive Care Guidelines for Management of Veno-Occlusive Disease in Children and Adolescents, Part 3: Focus on Cardiorespiratory Dysfunction, Infections, Liver Dysfunction, and Delirium. *Biol Blood Marrow Transplant* 2018; 24:207-218

203. Remy KE, Hall MW, Cholette J, et al.: Mechanisms of red blood cell transfusion-related immunomodulation. *Transfusion* 2018; 58:804-815

204. Rowan CM, Gertz SJ, Zinter MS, et al.: A multicenter investigation of respiratory syncytial viral infection in children with hematopoietic cell transplantation. *Transpl Infect Dis* 2018; 20:e12882

205. Rowan CM, Loomis A, McArthur J, et al.: High-frequency oscillatory ventilation use and severe pediatric ards in the pediatric hematopoietic cell transplant recipient. *Respiratory Care* 2018; 63:404-411

206. Rowan CM, McArthur J, Hsing DD, et al.: Acute Respiratory Failure in Pediatric Hematopoietic Cell Transplantation: A Multicenter Study. *Critical care medicine* 2018; 46:e967-e974

207. Shah S, Coppolino K, Menocha S, et al.: Immunomodulatory effects of plasma products on monocyte function in vitro. *J Trauma Acute Care Surg* 2018; 84:S47-s53

208. Steffen KM, Bateman ST, Valentine SL, et al.: Implementation of the Recommendations for RBC Transfusions for Critically Ill Children From the Pediatric Critical Care Transfusion and Anemia Expertise Initiative. *Pediatr Crit Care Med* 2018; 19:S170-s176

209. Steiner ME, Zantek ND, Stanworth SJ, et al.: Recommendations on RBC Transfusion Support in Children With Hematologic and Oncologic Diagnoses From the Pediatric Critical Care Transfusion and Anemia Expertise Initiative. *Pediatr Crit Care Med* 2018; 19:S149-s156

210. Tasker RC, Turgeon AF, Spinella PC: Recommendations on RBC Transfusion in Critically Ill Children With Acute Brain Injury From the Pediatric Critical Care Transfusion and Anemia Expertise Initiative. *Pediatr Crit Care Med* 2018; 19:S133-s136

211. Thomas NJ, Spear D, Wasserman E, et al.: CALIPSO: A Randomized Controlled Trial of Calfactant for Acute Lung Injury in Pediatric Stem Cell and Oncology Patients. *Biol Blood Marrow Transplant* 2018; 24:2479-2486

212. Tucci M, Lacroix J, Fergusson D, et al.: The age of blood in pediatric intensive care units (ABC PICU): study protocol for a randomized controlled trial. *Trials* 2018; 19:404

213. Valentine SL, Bembea MM, Muszynski JA, et al.: Consensus Recommendations for RBC Transfusion Practice in Critically Ill Children From the Pediatric Critical Care Transfusion and Anemia Expertise Initiative. *Pediatr Crit Care Med* 2018; 19:884-898

214. Watson RS, Asaro LA, Hertzog JH, et al.: Long-Term Outcomes after Protocolized Sedation versus Usual Care in Ventilated Pediatric Patients. *Am J Respir Crit Care Med* 2018; 197:1457-1467

215. Willson DF, Hall M, Beardsley A, et al.: Pediatric Ventilator-Associated Events: Analysis of the Pediatric Ventilator-Associated Infection Data. *Pediatr Crit Care Med* 2018; 19:e631-e636

216. Zantek ND, Parker RI, van de Watering LM, et al.: Recommendations on Selection and Processing of RBC Components for Pediatric Patients From the Pediatric Critical Care Transfusion and Anemia Expertise Initiative. *Pediatr Crit Care Med* 2018; 19:S163-s169

217. Best KM, Asaro LA, Curley MAQ, et al.: Sedation Management for Critically Ill Children with Pre-Existing Cognitive Impairment. *J Pediatr* 2019; 206:204-211 e201

218. Faustino EVS, Hirshberg EL, Asaro LA, et al.: Short-Term Adverse Outcomes Associated With Hypoglycemia in Critically Ill Children. *Crit Care Med* 2019; 47:706-714

219. Flori H, Sapru A, Quasney MW, et al.: A prospective investigation of interleukin-8 levels in pediatric acute respiratory failure and acute respiratory distress syndrome. *Crit Care* 2019; 23:128

220. Horak RV, Griffin JF, Brown AM, et al.: Growth and Changing Characteristics of Pediatric Intensive Care 2001-2016. *Crit Care Med* 2019; 47:1135-1142

221. Karam O, Nellis ME, Zantek ND, et al.: Criteria for Clinically Relevant Bleeding in Critically Ill Children: An International Survey. *Pediatr Crit Care Med* 2019; 20:e137-e144

222. Khemani RG, Smith L, Lopez-Fernandez YM, et al.: Paediatric acute respiratory distress syndrome incidence and epidemiology (PARDIE): an international, observational study. *Lancet Respir Med* 2019; 7:115-128

223. Lee JH, Nuthall G, Ikeyama T, et al.: Tracheal Intubation Practice and Safety Across International PICUs: A Report From National Emergency Airway Registry for Children. *Pediatr Crit Care Med* 2019; 20:1-8

224. Levy ER, Yip WK, Super M, et al.: Evaluation of Mannose Binding Lectin Gene Variants in Pediatric Influenza Virus-Related Critical Illness. *Front Immunol* 2019; 10:1005

225. Mahadeo KM, Khazal SJ, Abdel-Azim H, et al.: Management guidelines for paediatric patients receiving chimeric antigen receptor T cell therapy. *Nat Rev Clin Oncol* 2019; 16:45-63

226. Marsillio LE, Asaro LA, Srinivasan V, et al.: Outcomes Associated With Multiple Organ Dysfunction Syndrome in Critically Ill Children With Hyperglycemia. *Pediatr Crit Care Med* 2019; 20:1147-1156

227. Mokhateb-Rafii T, Bakar A, Gangadharan S, et al.: Hemodynamic Impact of Oxygen Desaturation During Tracheal Intubation Among Critically Ill Children With Cyanotic and Noncyanotic Heart Disease. *Pediatr Crit Care Med* 2019; 20:19-26

228. Napolitano N, Laverriere EK, Craig N, et al.: Apneic Oxygenation As a Quality Improvement Intervention in an Academic PICU. *Pediatr Crit Care Med* 2019; 20:e531-e537

229. Nellis ME, Dalton H, Karam O, et al.: Quantifiable Bleeding in Children Supported by Extracorporeal Membrane Oxygenation and Outcome. *Crit Care Med* 2019; 47:e886-e892

230. Nellis ME, Goel R, Karam O, et al.: International Study of the Epidemiology of Platelet Transfusions in Critically Ill Children with an Underlying Oncologic Diagnosis. *Pediatric Critical Care Medicine* 2019; 20:e342-e351

231. Nellis ME, Goel R, Karam O, et al.: Effects of ABO Matching of Platelet Transfusions in Critically Ill Children. *Pediatr Crit Care Med* 2019; 20:e61-e69

232. Nellis ME, Tucci M, Lacroix J, et al.: Bleeding Assessment Scale in Critically Ill Children (BASIC): Physician-Driven Diagnostic Criteria for Bleeding Severity. *Crit Care Med* 2019; 47:1766-1772

233. Randolph AG, Xu R, Novak T, et al.: Vancomycin Monotherapy May Be Insufficient to Treat Methicillin-resistant Staphylococcus aureus Coinfection in Children With Influenza-related Critical Illness. *Clin Infect Dis* 2019; 68:365-372

234. Shein SL, Karam O, Beardsley A, et al.: Development of an Antibiotic Guideline for Children With Suspected Ventilator-Associated Infections. *Pediatr Crit Care Med* 2019; 20:697-706

235. Spinella PC, Tucci M, Fergusson DA, et al.: Effect of Fresh vs Standard-issue Red Blood Cell Transfusions on Multiple Organ Dysfunction Syndrome in Critically Ill Pediatric Patients: A Randomized Clinical Trial. *JAMA* 2019; 322:2179-2190

236. Thakkar RK, Weiss SL, Fitzgerald JC, et al.: Risk Factors for Mortality in Pediatric Postsurgical versus Medical Severe Sepsis. *Journal of Surgical Research* 2019; 242:100-110

237. Treble-Barna A, Beers SR, Houtrow AJ, et al.: PICU-Based Rehabilitation and Outcomes Assessment: A Survey of Pediatric Critical Care Physicians. *Pediatr Crit Care Med* 2019; 20:e274-e282

238. Typpo K, Watson RS, Bennett TD, et al.: Outcomes of Day 1 Multiple Organ Dysfunction Syndrome in the PICU. *Pediatr Crit Care Med* 2019; 20:914-922

239. Watson RS, Asaro LA, Hutchins L, et al.: Risk Factors for Functional Decline and Impaired Quality of Life after Pediatric Respiratory Failure. *Am J Respir Crit Care Med* 2019; 200:900-909

240. Biagas KV, Hinton VJ, Hasbani NR, et al.: Long-Term Neurobehavioral and Quality of Life Outcomes of Critically Ill Children after Glycemic Control. *J Pediatr* 2020; 218:57-63.e55

241. Boyd DF, Allen EK, Randolph AG, et al.: Exuberant fibroblast activity compromises lung function via ADAMTS4. *Nature* 2020; 587:466-471

242. Branca A, Tellez D, Berkenbosch J, et al.: The New Trainee Effect in Tracheal Intubation Procedural Safety Across PICUs in North America: A Report From National Emergency Airway Registry for Children. *Pediatr Crit Care Med* 2020; 21:1042-1050

243. Conway JA, Kharayat P, Sanders RC, Jr., et al.: Ketamine Use for Tracheal Intubation in Critically Ill Children Is Associated With a Lower Occurrence of Adverse Hemodynamic Events. *Crit Care Med* 2020; 48:e489-e497

244. Dahmer MK, Flori H, Sapru A, et al.: Surfactant Protein D Is Associated With Severe Pediatric ARDS, Prolonged Ventilation, and Death in Children With Acute Respiratory Failure. *Chest* 2020; 158:1027-1035

245. Daigle CH, Fiadjoe JE, Laverriere EK, et al.: Difficult Bag-Mask Ventilation in Critically Ill Children Is Independently Associated With Adverse Events. *Crit Care Med* 2020; 48:e744-e752

246. Demaret P, Karam O, Labreuche Bst J, et al.: How 217 Pediatric Intensivists Manage Anemia at PICU Discharge: Online Responses to an International Survey. *Pediatr Crit Care Med* 2020; 21:e342-e353

247. Emeriaud G, Napolitano N, Polikoff L, et al.: Impact of Failure of Noninvasive Ventilation on the Safety of Pediatric Tracheal Intubation. *Crit Care Med* 2020; 48:1503-1512

248. Feldstein LR, Rose EB, Horwitz SM, et al.: Multisystem Inflammatory Syndrome in U.S. Children and Adolescents. *N Engl J Med* 2020; 383:334-346

249. Fink EL, Jarvis JM, Maddux AB, et al.: Development of a core outcome set for pediatric critical care outcomes research. *Contemp Clin Trials* 2020; 91:105968

250. Fink EL, Maddux AB, Pinto N, et al.: A Core Outcome Set for Pediatric Critical Care. *Crit Care Med* 2020; 48:1819-1828

251. Gertz SJ, McArthur J, Hsing DD, et al.: Respiratory pathogens associated with intubated pediatric patients following hematopoietic cell transplant. *Transplant Infectious Disease* 2020; 22

252. Keim G, Yehya N, Spear D, et al.: Development of Persistent Respiratory Morbidity in Previously Healthy Children After Acute Respiratory Failure. *Crit Care Med* 2020; 48:1120-1128

253. Kneyber MCJ, Cheifetz IM, Curley MAQ: High-frequency oscillatory ventilation for PARDS: awaiting PROSPect. *Crit Care* 2020; 24:118

254. Kudchadkar SR, Nelliot A, Awojoodu R, et al.: Physical Rehabilitation in Critically Ill Children: A Multicenter Point Prevalence Study in the United States. *Critical Care Medicine* 2020:634-644

255. LaMarra D, French J, Bailey C, et al.: A Novel Framework Using Remote Telesimulation With Standardized Parents to Improve Research Staff Preparedness for Informed Consent in Pediatric Critical Care Research. *Pediatr Crit Care Med* 2020; 21:e1042-e1051

256. Lincoln PA, Whelan K, Hartwell LP, et al.: Nurse-Implemented Goal-Directed Strategy to Improve Pain and Sedation Management in a Pediatric Cardiac ICU. *Pediatr Crit Care Med* 2020; 21:1064-1070

257. López-Fernández YM, Smith LS, Kohne JG, et al.: Prognostic relevance and inter-observer reliability of chest-imaging in pediatric ARDS: a pediatric acute respiratory distress incidence and epidemiology (PARDIE) study. *Intensive Care Medicine* 2020; 46:1382-1393

258. Maddux AB, Pinto N, Fink EL, et al.: Postdischarge Outcome Domains in Pediatric Critical Care and the Instruments Used to Evaluate Them: A Scoping Review. *Crit Care Med* 2020; 48:e1313-e1321

259. Mahadeo KM, Bajwa R, Abdel-Azim H, et al.: Diagnosis, grading, and treatment recommendations for children, adolescents, and young adults with sinusoidal obstructive syndrome: an international expert position statement. *Lancet Haematol* 2020; 7:e61-e72

260. Miller AG, Napolitano N, Turner DA, et al.: Respiratory Therapist Intubation Practice in Pediatric ICUs: A Multicenter Registry Study. *Respir Care* 2020; 65:1534-1540

261. Nellis ME, Saini A, Spinella PC, et al.: Pediatric plasma and platelet transfusions on extracorporeal membrane oxygenation: A subgroup analysis of two large international point-prevalence studies and the role of local guidelines. *Pediatric Critical Care Medicine* 2020:267-275

262. Nellis ME, Spinella PC, Tucci M, et al.: Effect of platelet storage duration on clinical outcomes and incremental platelet change in critically ill children. *Transfusion* 2020; 60:2849-2858

263. Noël KC, Papenburg J, Lacroix J, et al.: International Survey on Determinants of Antibiotic Duration and Discontinuation in Pediatric Critically Ill Patients. *Pediatr Crit Care Med* 2020; 21:e696-e706

264. Novak T, Hall MW, McDonald DR, et al.: RIG-I and TLR4 responses and adverse outcomes in pediatric influenza-related critical illness. *J Allergy Clin Immunol* 2020; 145:1673-1680.e1611

265. Rowan CM, Klein MJ, Hsing DD, et al.: Early Use of Adjunctive Therapies for Pediatric Acute Respiratory Distress Syndrome: A PARDIE Study. *Am J Respir Crit Care Med* 2020; 201:1389-1397

266. Sauthier MS, Jouvet PA, Newhams MM, et al.: Machine Learning Predicts Prolonged Acute Hypoxemic Respiratory Failure in Pediatric Severe Influenza. *Crit Care Explor* 2020; 2:e0175

267. Srinivasan V, Hasbani NR, Mehta NM, et al.: Early Enteral Nutrition Is Associated With Improved Clinical Outcomes in Critically Ill Children: A Secondary Analysis of Nutrition Support in the Heart and Lung Failure-Pediatric Insulin Titration Trial. *Pediatr Crit Care Med* 2020; 21:213-221

268. Steppan DA, Coleman RD, Viamonte HK, et al.: Outcomes of pediatric patients with oncologic disease or following hematopoietic stem cell transplant supported on extracorporeal membrane oxygenation: The PEDECOR experience. *Pediatr Blood Cancer* 2020; 67:e28403

269. Ward SL, Dahmer MK, Weeks HM, et al.: Association of patient weight status with plasma surfactant protein D, a biomarker of alveolar epithelial injury, in children with acute respiratory failure. *Pediatr Pulmonol* 2020; 55:2730-2736

270. Ward SL, Flori HR, Bennett TD, et al.: Design and Rationale for Common Data Elements for Clinical Research in Pediatric Critical Care Medicine. *Pediatr Crit Care Med* 2020; 21:e1038-e1041

271. Weiss SL, Peters MJ, Alhazzani W, et al.: Surviving sepsis campaign international guidelines for the management of septic shock and sepsis-associated organ dysfunction in children. *Intensive Care Med* 2020; 46:10-67

272. Yehya N, Harhay MO, Klein MJ, et al.: Predicting Mortality in Children with Pediatric Acute Respiratory Distress Syndrome: A Pediatric Acute Respiratory Distress Syndrome Incidence and Epidemiology Study. *Critical Care Medicine* 2020:E514-E522

273. Bhalla AK, Klein MJ, Emeriaud G, et al.: Adherence to Lung-Protective Ventilation Principles in Pediatric Acute Respiratory Distress Syndrome: A Pediatric Acute Respiratory Distress Syndrome Incidence and Epidemiology Study. *Crit Care Med* 2021; 49:1779-1789

274. Bridges BC, Kilbaugh TJ, Barbaro RP, et al.: Veno-Venous Extracorporeal Membrane Oxygenation for Children With Cancer or Hematopoietic Cell Transplant: A Ten Center Cohort. *Asaio j* 2021; 67:923-929

275. Burton CL, Furlong-Dillard JM, Jawad K, et al.: Analysis of Viscoelastic Testing in Pediatric Patients Using the Pediatric Extracorporeal Membrane Oxygenation Outcomes Registry. *ASAIO J* 2021; 67:1251-1256

276. Carlton EF, Donnelly JP, Prescott HC, et al.: School and Work Absences After Critical Care Hospitalization for Pediatric Acute Respiratory Failure: A Secondary Analysis of a Cluster Randomized Trial. *JAMA Netw Open* 2021; 4:e2140732

277. Carlton EF, Pinto N, Smith M, et al.: Overall Health Following Pediatric Critical Illness: A Scoping Review of Instruments and Methodology. *Pediatr Crit Care Med* 2021

278. Faustino EVS, Raffini LJ, Hanson SJ, et al.: Age-Dependent Heterogeneity in the Efficacy of Prophylaxis With Enoxaparin Against Catheter-Associated Thrombosis in Critically Ill Children: A Post Hoc Analysis of a Bayesian Phase 2b Randomized Clinical Trial. *Crit Care Med* 2021; 49:e369-e380

279. Faustino EVS, Shabanova V, Raffini LJ, et al.: Efficacy of Early Prophylaxis Against Catheter-Associated Thrombosis in Critically Ill Children: A Bayesian Phase 2b Randomized Clinical Trial. *Crit Care Med* 2021; 49:e235-e246

280. Feldstein LR, Self WH, Ferdinands JM, et al.: Incorporating Real-time Influenza Detection Into the Test-negative Design for Estimating Influenza Vaccine Effectiveness: The Real-time Test-negative Design (rtTND). *Clin Infect Dis* 2021; 72:1669-1675

281. Feldstein LR, Tenforde MW, Friedman KG, et al.: Characteristics and Outcomes of US Children and Adolescents with Multisystem Inflammatory Syndrome in Children (MIS-C) Compared with Severe Acute COVID-19. *JAMA - Journal of the American Medical Association* 2021; 325:1074-1087

282. Geva A, Patel MM, Newhams MM, et al.: Data-driven clustering identifies features distinguishing multisystem inflammatory syndrome from acute COVID-19 in children and adolescents. *EClinicalMedicine* 2021; 40:101112

283. Ghafoor S, Fan K, Di Nardo M, et al.: Extracorporeal Membrane Oxygenation Candidacy in Pediatric Patients Treated With Hematopoietic Stem Cell Transplant and Chimeric Antigen Receptor T-Cell Therapy: An International Survey. *Front Oncol* 2021; 11:798236

284. Hirshberg EL, Alexander JL, Asaro LA, et al.: Performance of an Electronic Decision Support System as a Therapeutic Intervention During a Multicenter PICU Clinical Trial: Heart and Lung Failure-Pediatric Insulin Titration Trial (HALF-PINT). *Chest* 2021; 160:919-928

285. Jaber B, Bembea MM, Loftis LL, et al.: Venovenous Versus Venoarterial Extracorporeal Membranous Oxygenation in Inotrope Dependent Pediatric Patients With Respiratory Failure. *Asaio j* 2021; 67:457-462

286. Kachmar AG, Wypij D, Perry MA, et al.: Income-driven socioeconomic status and presenting illness severity in children with acute respiratory failure. *Res Nurs Health* 2021; 44:920-930

287. Karsies T, Tarquinio K, Shein SL, et al.: Compliance With an Antibiotic Guideline for Suspected Ventilator-Associated Infection: The Ventilator-Associated INfection (VAIN2) Study. *Pediatr Crit Care Med* 2021; 22:859-869

288. Killien EY, Loftis LL, Clark JD, et al.: Health-related quality of life outcome measures for children surviving critical care: a scoping review. *Qual Life Res* 2021; 30:3383-3394

289. Kopp W, Gedeit RG, Asaro LA, et al.: The Impact of Preintubation Noninvasive Ventilation on Outcomes in Pediatric Acute Respiratory Distress Syndrome. *Crit Care Med* 2021; 49:816-827

290. Larovere KL, Riggs BJ, Poussaint TY, et al.: Neurologic Involvement in Children and Adolescents Hospitalized in the United States for COVID-19 or Multisystem Inflammatory Syndrome. *JAMA Neurology* 2021:E1-E12

291. Maue DK, Mangus RS, Lutfi R, et al.: Practice variation in the immediate postoperative care of pediatric liver transplant patients: Framework for a national consensus. *Pediatr Transplant* 2021; 25:e13976

292. Mbf S, N M, K F, et al.: Multisystem Inflammatory Syndrome in Children - Initial Therapy and Outcomes. *The New England journal of medicine* 2021; 385:23-34

293. Monteiro ACC, Flori H, Dahmer MK, et al.: Thrombomodulin is associated with increased mortality and organ failure in mechanically ventilated children with acute respiratory failure: biomarker analysis from a multicenter randomized controlled trial. *Crit Care* 2021; 25:271

294. Muttalib F, González-Dambrauskas S, Lee JH, et al.: Pediatric Emergency and Critical Care Resources and Infrastructure in Resource-Limited Settings: A Multicountry Survey. *Crit Care Med* 2021; 49:671-681

295. Natale JE, Asaro LA, Joseph JG, et al.: Association of Race and Ethnicity with Sedation Management in Pediatric Intensive Care. *Ann Am Thorac Soc* 2021; 18:93-102

296. Nishisaki A, Lee A, Li S, et al.: Sustained Improvement in Tracheal Intubation Safety Across a 15-Center Quality-Improvement Collaborative: An Interventional Study From the National Emergency Airway Registry for Children Investigators. *Crit Care Med* 2021; 49:250-260

297. Olson SM, Newhams MM, Halasa NB, et al.: Effectiveness of Pfizer-BioNTech mRNA Vaccination Against COVID-19 Hospitalization Among Persons Aged 12-18 Years - United States, June-September 2021. *MMWR Morb Mortal Wkly Rep* 2021; 70:1483-1488

298. Ozment CP, Scott BL, Bembea MM, et al.: Anticoagulation and Transfusion Management During Neonatal and Pediatric Extracorporeal Membrane Oxygenation: A Survey of Medical Directors in the United States. *Pediatr Crit Care Med* 2021; 22:530-541

299. Payne AB, Gilani Z, Godfred-Cato S, et al.: Incidence of Multisystem Inflammatory Syndrome in Children Among US Persons Infected With SARS-CoV-2. *JAMA Netw Open* 2021; 4:e2116420

300. Perry MA, Dawkins-Henry OS, Awojoodu RE, et al.: Study protocol for a two-center test of a nurse-implemented chronotherapeutic restoring bundle in critically ill children: RESTORE Resilience (R(2)). *Contemp Clin Trials Commun* 2021; 23:100840

301. Pinto VL, Guffey D, Loftis L, et al.: Evaluation of Severity of Illness Scores in the Pediatric ECMO Population. *Front Pediatr* 2021; 9:698120

302. Ragoonanan D, Khazal SJ, Abdel-Azim H, et al.: Diagnosis, grading and management of toxicities from immunotherapies in children, adolescents and young adults with cancer. *Nat Rev Clin Oncol* 2021; 18:435-453

303. Rajapreyar P, Castaneda L, Thompson NE, et al.: Association of Fluid Balance and Survival of Pediatric Patients Treated With Extracorporeal Membrane Oxygenation. *Front Pediatr* 2021; 9:722477

**Supplemental Table 1: Journals of PALISI Publications**

| **Rank** | **Journal Name** | **Number of Articles** |
| --- | --- | --- |
| 1 | Pediatric Critical Care Medicine | 131 |
| 2 | Critical Care Medicine | 36 |
| 3 | Journal of Pediatric Intensive Care | 11 |
| 4 | Transfusion | 10 |
| 5 | American Journal of Respiratory and Critical Care Medicine | 9 |
| 6 | JAMA | 6 |
| 7 | Biology of Blood and Marrow Transplantation | 5 |
| 7 | Critical Care | 5 |
| 7 | Journal of Pediatrics | 5 |
| 8 | Frontiers in Pediatrics | 4 |
| 8 | Intensive Care Med | 4 |
| 8 | NEJM | 4 |
| 8 | Pediatrics | 4 |
| 9 | ASAIO | 3 |
| 9 | Chest | 3 |
| 9 | Contemporary Clinical Trials | 3 |
| 9 | Journal of Infectious Diseases | 3 |
| 10 | Annals of Intensive Care | 2 |
| 10 | Annals of thoracic surgery | 2 |
| 10 | Clinical Infectious Diseases | 2 |
| 10 | Frontiers in Immunology | 2 |
| 10 | Jama Open Network | 2 |
| 10 | Journal of Diabetes Science and Technology | 2 |
| 10 | Nature Reviews Clinical Oncology | 2 |
| 10 | Pediatric Pulmonology | 2 |
| 10 | Respiratory Care | 2 |
| 10 | Transplant Infectious Disease | 2 |
| 10 | Trials | 2 |
| 11 | American Journal of Medical Quality | 1 |
| 11 | An Pediatr (Barc.) | 1 |
| 11 | Annals of ATS | 1 |
| 11 | Annals of Surgery | 1 |
| 11 | BMC Pediatrics | 1 |
| 11 | Bone Marrow Transplantation | 1 |
| 11 | Cardiology in the Young | 1 |
| 11 | Contemporary Clinical Trials Communications | 1 |
| 11 | Critical Care Explorations | 1 |
| 11 | EClinical Medicine | 1 |

**Supplemental Table 1: Journals of PALISI Publications**

| 11 | Frontiers in Oncology | 1 |
| --- | --- | --- |
| 11 | Heart & Lung | 1 |
| 11 | JAMA Neurology | 1 |
| 11 | JAMA Peds | 1 |
| 11 | Journal of Allergy and Clinical Immunology | 1 |
| 11 | Journal of Biomedical Informatics | 1 |
| 11 | Journal of Critical Care | 1 |
| 11 | Journal of Surgical Research | 1 |
| 11 | Journal of Trauma and Acute Care Surgery | 1 |
| 11 | Lancet Respiratory Medicine | 1 |
| 11 | medicine sciences | 1 |
| 11 | MMWR | 1 |
| 11 | Nature | 1 |
| 11 | Nature Medicine | 1 |
| 11 | Nursing Research Online | 1 |
| 11 | Pediatric Blood and Cancer | 1 |
| 11 | Pediatric Transplantation | 1 |
| 11 | Quality of Life Research | 1 |
| 11 | Research in Nursing and Health | 1 |
| 11 | The Lancet | 1 |
| 11 | The Lancet Hematology | 1 |
| 11 | Transfusion Clinique et Biologique | 1 |
| 11 | Transfusion Practice | 1 |
| 11 | Transplantation Proceedings | 1 |
| 11 | Vox Sanguinis | 1 |
